# Diagnosing dehydrated hereditary stomatocytosis due to a *KCNN4* Gardos channel mutation: understanding challenges through study of a multi‐generational family

**DOI:** 10.1002/jha2.267

**Published:** 2021-07-22

**Authors:** Sasha Waldstein, Sarah Arnold‐Croop, Laura Carrel, M. Elaine Eyster

**Affiliations:** ^1^ Division of Hematology/Oncology Penn State Hershey Medical Center Hershey Pennsylvania USA; ^2^ Department of Biochemistry and Molecular Biology Penn State College of Medicine Hershey Pennsylvania USA

**Keywords:** hereditary anemia, iron overload, non‐spherocytic hemolytic anemia

To the Editor,

Dehydrated Hereditary Stomatocytosis (DHSt) is autosomal dominant hemolytic anemia with an estimated incidence of 1:50,000 births. DHSt is characterized by largely compensated hemolysis and splenomegaly and is attributed primarily to mutations in either the *PIEZO1* or *KCNN4* genes [1]. The majority of described DHSt cases result from gain‐of‐function mutations in the mechanosensitive cation channel gene *PIEZO1*, leading to an inappropriate increase in calcium influx. This, in turn, activates the calcium‐sensitive, potassium‐selective Gardos channel which mediates potassium conductance and regulates red cell volume [2]. Less frequently, gain‐of‐function mutations have been identified in the Gardos channel gene, *KCNN4*, that alter calcium sensitivity and result in a more active channel. Therefore, DHSt due to *KCNN4* mutation is aptly termed a Gardos channelopathy [2, 3, 4]. Though a complex compensatory mechanism has been proposed in those with *KCNN4* mutations, the increased cation leak across the red blood cell membrane in cases with either *PIEZO1* or *KCNN4* mutations is accompanied by intracellular dehydration and the formation of stomatocytes [4].

Since the first reports in 2015, to our knowledge, *KCNN4* mutations have been identified in ten DHSt families [2, 3, 4, 5, 6]. *PIEZO1* mutations in DHSt are more prevalent, with at least eight times as many cases identified [2]. Here we report the eleventh family with DHSt due to a *KCNN4* substitution mutation that results in p.R352H. This family highlights the barriers to diagnosis, which include the variable phenotype, paucity of characteristic xerocytes and stomatocytes on peripheral smear, and omission of *KCNN4* on many commercially available hemolytic anemia panels. The correct diagnosis was made only after in‐house whole‐exome sequencing was performed. Recognition and appropriate diagnosis of this disease are critical to better understand the true prevalence and clinical phenotype of individuals carrying *KCNN4* mutations and to facilitate the development of new treatments.

Five subjects, from a single US Caucasian family of European descent with no extended history of jaundice or anemia, were enrolled in this study after International Review Board (IRB) approval and informed consent. The subjects included the proband, her unaffected husband, her two children (both affected), and an unaffected grandchild. The proband was jaundiced, with laboratory studies consistent with hemolytic anemia at birth (Table 1). She had normal growth and development, but underwent a splenectomy at 3 years of age for a chronic “non‐spherocytic hemolytic anemia.” Her jaundice and anemia persisted following splenectomy. At the age of 8, she underwent cholecystectomy for cholelithiasis. She has maintained average hemoglobin of 10.5 g/dL with no transfusion requirements, other than during a delivery complicated by placental abruption. At age 61, almost 58 years post‐splenectomy, she has experienced no thrombotic complications. Prior to enrollment in this study, she had an extensive evaluation of her hemolytic anemia at multiple institutions. Peripheral smear showed anisopoikilocytosis with hypochromia and stippling, polychromatophilia, occasional Howell‐Jolly bodies, and few stomatocytes. Other than a mildly elevated red cell sodium of 19.4 mEq/L and a reported small population of cells with decreased osmotic fragility after incubation, there were no notable abnormalities to lead to a definitive diagnosis.

**TABLE 1:** Hematologic data for proband, son, and daughter.^a^

	**Age**	**Hgb (13–17 g/dl)**	**MCV (81–96 fl)**	**MCHC (32–36 g/dl)**	**RDW (11.5–14.2%)**	**Retic count (0.4–2%)**	**Platelet count (150–350 K/μl)**	**Total bilirubin (0–1.2 mg/dl)**	**Ferritin F (13–150 ng/ml) M (30–400 ng/ml)**	**LDH (100—250 u/L)**
Proband^b^, ^c^	61	10.5 (±0.3)	106.5 (±1.3)	34.1 (±0.5)	15 (±0.4)	12.6 (±2)	532.2 (±32.9)	6 (±0.9) (Bili D < 0.1 ‐ 0.6)	503.2 (±113.2)	438.5 (±153)
Daughter	39	11.1 (±0.7)	99.9 (±2.9)	33.9 (±1)	16.1 (±0.3)	6.7 (±1.5)	192.3 (±49.4)	2.3 (±0.5) (Bili D < 0.1‐ 0.3)	222.5 (±84.5)	301 (±148)
Son^b^, ^d^	34	9.3 (±0.4)	98.8 (±3.1)	32.9 (±0.7)	21.2 (±0.6)	>22	837 (±64.7)	5.7 (±0.7) (Bili D < 0.1 – 0.4)	1061.9 (±53.5)	483.5 (±233.5)

^a^ Means calculated from at least 25 measurements, except for Ferritin and LDH which are means of at least four values.

^b^ Splenectomized.

^c^ Post‐menopausal. Prior to menopause, her average ferritin was 369 ng/ml (±109.8).

^d^ Ferritin values prior to iron chelation therapy.

The proband's daughter has had mild persistent hemolytic anemia since birth. Her peripheral blood smear showed polychromasia, few stomatocytes, target cells, and few fragments. At age 39, she has mild splenomegaly and no gallstones. Her 4‐year‐old daughter is not affected and had normal hemoglobin of 11.8 g/dl at 1.5 years of age.

The proband's son, age 34, has more severe hemolytic anemia and hyperbilirubinemia, with an average hemoglobin value of 9.3 g/dl, but has never required transfusions. He had a cholecystectomy at age four for cholelithiasis. He had splenomegaly, and a splenectomy was performed as a teenager for immune thrombocytopenia. At age 32, he developed iron overload, prompting initiation of iron chelation therapy with deferasirox for maximum ferritin of 1132 ng/ml and iron saturation > 100%. In the approximately 20 years since splenectomy, he has had no thrombotic complications.

A targeted Next Generation Hereditary Hemolytic Anemia sequencing panel performed on the proband by an outside reference laboratory revealed that she was homozygous for the UGT1A1*28 (TA7) promoter allele, consistent with Gilbert disease. While this contributed to her hyperbilirubinemia, it did not explain the hemolytic anemia. In addition, she was heterozygous for three variants of uncertain significance in the *FANCC*, *HMOX1*, and *SPTA1* genes. This panel did not include testing for *KCNN4*. To provide a definitive diagnosis, exome sequencing was performed on the three affected subjects and the proband's unaffected husband and granddaughter. Targeted analysis was restricted to 24 genes known to encode red cell cytoskeletal membrane or channel proteins that had not been included in the panel from the reference laboratory. Genes screened included: ATP binding cassette subfamily G members (*ABCG5* and *ABCG8*), ATPase phospholipid transporting 11c (*ATP11c*), collagen type IV alpha 1 chain (*COL4A1*), erythrocyte membrane protein band 4.1 (*EPB41*), potassium calcium‐activated channel subfamily N member 4 (*KCNN4*), UDP glucuronosyltransferase family 1 member A6 and A7 (*UGT1A6*, *UGTA17*), X‐linked Kx blood group (*XK*), glutathione peroxidase 1 (*GPX1)*, glycophorin A (*GYPA*), glycophorin B (*GYPB*), CD47, adducin 1 (*ADD1*), adducin 2 (*ADD2*), atypical chemokine receptor 1 (*ACKR1*), ankyrin 1 (*ANK1*), dematin actin‐binding protein (*DMTN*), kell system (*KEL*), intercellular adhesion molecule 4 (*ICAM4*), peroxiredoxin 2 (*PRDX2*), erythrocyte tropomodulin (*TMOD1)*, tropomyosin 1 (*TPM1*), TRIO, and F‐Actin binding protein (*TRIOBP*). The only known pathogenic mutation identified was a missense mutation in *KCNN4* that results in the substitution p.R352H. This mutation was found in all three affected family members and was absent in unaffected individuals.

Previous clinical reports highlight phenotypic variability both within and between the ten *KCNN4* families [2, 3, 4, 5, 6]. It is possible that some differences are due to the specific underlying mutation; seven families have p.R352H, whereas the other families had novel mutations of p.V282E, p.V282M, and a 28 bp deletion (c.1109_1119 + 17del). One large family with the p.V282M mutation had well‐compensated anemia with near‐normal hemoglobin levels [5]. In contrast, the proband of another family with p.R352H required transfusion in utero and after preterm birth [3]. Other cases, including those with p.R352H, had variable transfusion requirements during childhood, typically without the ongoing need for transfusion support during adulthood. The majority developed cholelithiasis requiring cholecystectomy. Iron overload was relatively common, though variable within and between families. As in our family, many reported cases underwent splenectomy before a formal diagnosis was made, and splenectomy failed to improve the disease features [2, 5].

Although the small numbers of reported DHSt cases, especially with *KCNN4* mutations, limit conclusions, differences between the phenotypes of *PIEZO1* versus *KCNN4* gene mutations are emerging [2, 3, 4, 5, 6]. This is particularly the case with regard to the frequency of iron overload, regardless of transfusion regimen, and post‐splenectomy venous thromboses. In a retrospective review by Picard, et al. of 126 patients, including twelve from six *KCNN4* families, the mean ferritin in *KCNN4* patients at diagnosis was 1702 ng/ml, compared to 656 ng/ml for patients with *PIEZO1* mutations [2]. Notably, all eight splenectomized *PIEZ01* patients in that review developed subsequent thrombotic events requiring long‐term anticoagulation. In contrast, none of the four reported splenectomized *KCNN4* patients experienced thrombotic events, despite a mean of 26.5 years post‐splenectomy. Other case reports with long‐term follow‐up post‐splenectomy, including the one presented here, further support this difference between the hypercoagulable state post‐splenectomy for *PIEZO1* patients as compared to the absence of thrombotic risk in *KCNN4* mutated DHSt [2, 5]. Increased understanding of the phenotypic differences, only possible through appropriate diagnosis and reporting of DHSt that results from both *KCNN4* and *PIEZO1* mutations, is essential to ensure appropriate treatment of these patients.

The recognition and diagnosis of DHSt remain challenging. Not only are the phenotypic patterns different, both between and within *PIEZO1* and *KCNN4* mutations, but the defining laboratory values also differ. The characteristic findings of red cell dehydration, with an elevated mean corpuscular hemoglobin concentration (MCHC), decreased osmotic fragility, and a left‐shifted osmotic gradient ektacytometry, are often absent in patients with *KCNN4* mutations [2]. In the family reported here, as well as others, stomatocytes were infrequent, no xerocytes were identified to assist in diagnosis, and the MCHC was normal. Furthermore, the typical features described for the disease process of DHSt are largely influenced by the disparity in case numbers, which are heavily weighted toward *PIEZ01* mutations.

As DHSt due to a Gardos channel *KCNN4* mutation is likely to be much more prevalent than reported, confirmation is critical to counsel patients and families appropriately, avoid unnecessary splenectomy and recognize iron overload early in the course of this disorder. Increased recognition of this disease process could also lead to significant treatment advances, such as the use of the selective Gardos channel inhibitor, Senicapoc. This medication has been shown to improve hemolysis in patients with sickle cell disease and might be used to prevent red blood cell dehydration due to a gain of function mutation in *KCNN4* [1]. The recognition of this specific disease entity will require a high level of clinical suspicion, plus the inclusion of the *KCNN4* gene mutation in commercially available hemolytic anemia panels to appropriately investigate those patients with chronic non‐spherocytic hemolytic anemia.

## CONFLICT OF INTEREST

The authors have declared no conflict of interest.

## ETHICS APPROVAL

This study was performed with IRB approval (Study 00009292), informed consent, and ethics approval as specified by the Human Subjects Protection Office of the Milton S Hershey Medical Center of the Pennsylvania State University.

## Data Availability

Supplemental exome sequence data are available on reasonable request to Dr. Carrel.
